# Effects of subitems in the colorectal cancer screening protocol on the Chinese colorectal cancer screening program: an analysis based on natural community screening results

**DOI:** 10.1186/s12885-018-5191-y

**Published:** 2019-01-10

**Authors:** Shan-Rong Cai, Yan-Qin Huang, Su-Zhan Zhang, Qi-Long Li, Xin Yuan Ma, Shu Zheng

**Affiliations:** 1 Cancer Institute (Key Laboratory of Cancer Prevention and Intervention, Ministry of Education of China, Key Laboratory of Molecular Biology in Medical Sciences, Zhejiang Province, China), 88 Jiefang Rd, Hangzhou, 310009 People’s Republic of China; 2 The second Affiliated Hospital, School of Medicine, Zhejiang University, Hangzhou, Zhejiang 310009 People’s Republic of China; 3 Institute of Oncology Prevention and Treatment, Jia-Shan, Zhejiang, 314100 People’s Republic of China

**Keywords:** Colorectal cancer, Screening, Advanced neoplasm, Cost-effectiveness analysis

## Abstract

**Background:**

To date, no single colorectal cancer (CRC) screening strategy has been determined to be applicable worldwide. In China, a CRC screening protocol that combines double fecal immunochemical tests (FITs) and a high-risk factor questionnaire (HRFQ) as the first stage of screening and colonoscopy as the second stage of screening (scenario A) was adapted by the Chinese Ministry of Health in 2006. However, applying this CRC screening protocol nationally remains difficult because its effectiveness and convenience are controversial. This study evaluated the effects of subitems of the CRC screening protocol in China.

**Methods:**

CRC screening results (scenario A) from Jiashan County, China, (2007–2009) were used to analyze the detection rates of CRC and advanced neoplasms as well as the cost-effectiveness of the protocol. Scenario A was divided into scenarios B–G (by selecting some items at the first stage of screening) for analysis.

**Results:**

Compared with scenario A, removing the whole HRFQ (scenario F) reduced advanced neoplasm and adenoma detections by 29.8 and 41.2%, respectively, whereas the whole HRFQ accounted for 10.1% of the total screening cost. Removing FITs (scenario G) reduced CRC, advanced neoplasm and adenoma detections by 71.8, 56.9 and 47.7%, respectively, and the costs per case of CRC and advanced neoplasm were 82.0 and 19.1% higher, respectively, than those in scenario A.

In scenarios B–E (deleting some high-risk factor questions on the HRFQ), the odds ratios (ORs) of the detection rates and costs per CRC, advanced neoplasm, adenoma, and neoplasm case were near 1.00.

Scenarios C and D reduced the high-risk population and total screening costs by less than 6.0 and 4.1%, respectively. Scenarios E and B (FITs and a personal history of cancer or colorectal adenoma were reserved) reduced the high-risk population by 17.6 and 24.2% and the total screening costs by 11.2 and 15.4%, respectively, while the numbers of CRC cases were not missed, and advanced neoplasms detected decreased by only 5 and 11%, respectively.

**Conclusion:**

The results of this study demonstrate that FITs and a personal history of colorectal adenoma are the most effective items in the Chinese CRC screening protocol.

## Background

The incidences of colorectal cancer (CRC), breast cancer, and lung cancer have rapidly increased in recent years in China, and the cancer spectrum in China is becoming similar to that in Western developed countries, although China is still considered a developing country [1, 2]. CRC will soon impose a great public health burden in China and is currently a heavy burden in Western developed countries. Several randomized controlled trials (RCTs) have demonstrated that screening effectively reduces CRC morbidity and mortality [3–6]. The decrease in CRC mortality in the U.S.A. since 1998 is largely attributed to the popularity of CRC screening in recent decades [7–9]. At present, the CRC screening protocols reported and recommended by some experts and institutions include various methods, such as the fecal occult blood test (FOBT) plus colonoscopy, flexible sigmoidoscopy and colonoscopy [10, 11]. However, no single best CRC screening strategy exists globally. In China, a CRC screening protocol combining double fecal immunochemical tests (FITs) and a high-risk factor questionnaire (HRFQ) was adapted by the Chinese Ministry of Health in 2006 and phased into the national screening program [12]. However, this CRC screening protocol is difficult to apply globally because its effectiveness and convenience are controversial [13]. Therefore, the screening protocol should be re-evaluated by analyzing the cost-effectiveness of the protocol.

## Methods and samples

The entire resident population in three communities (Ganyao, Da yun and Yaozhuang) in Jiashan County, Zhejiang Province, China, and aged 40 to 74 years were invited to attend free CRC screening programs in 2007, 2008, and 2009, respectively, and 31,963 residents(aged 40 to 74 years old, males: 16,169; females: 15,794) from the entire population were defined as the targeted screening population.

All eligible participants who attended the screening program signed written consent forms. A two-stage screening design was used. FITs and the HRFQ were used as primary screening methods in the first stage. The FIT (qualitative FIT test kits were purchased from W.H.P.M., Inc., Beijing, China) was repeated twice with a one-week interval between tests. If one reader could not ensure the FIT positive results, then a second reader reviewed the results. If the results remained uncertain, we repeated the FIT tests to ensure the results were reliable.

HRFQ-positive was defined as follows: 1) individuals having one of the following events: a) history of cancer, b) history of colorectal adenoma, or c) family history of CRC in first-degree relatives, and/or 2) individuals having at least two of the following events: a) chronic coprostasis, b) chronic diarrhea, c) phlegmatically bloody feces, d) severely stressful life events among first-degree relatives, e) chronic appendicitis or appendectomy, and f) chronic cholecystitis or cholecystectomy. we trained the interviewer well before the screening and did quality control by telephone investigation with epidemiological researchers one week after the first investigation was completed. In addition, we created a manual and delivered this guide to each investigator to ensure the quality of the investigation.

If either the FIT or the HRFQ was positive, a colonoscopy was recommended in the second stage. If the colonoscopy showed a positive result, a biopsy and histopathological diagnosis were performed after the patient signed the consent form and paid for a biopsy and histopathological exam. This two-step screening protocol was accepted by the National Cancer Screening Program in 2006. All cancer cases were confirmed by the Jiashan Cancer Registration System with histopathological diagnosis. Based on the international classification, CRC was defined as the invasion of malignant cells beyond the muscular mucosa. Patients with intramucosal carcinoma or carcinoma in situ were classified as having high-grade dysplasia. Pathologic slides of positive lesions were re-examined and diagnosed by consensus from at least two independent pathologists.

### Statistical analysis

The Statistical Package for the Social Sciences (SPSS) software, version 21.0 was used to analyze the data. Differences in proportions are listed in the tables, and the odds ratio (OR) was used to compare the detection rates between the national screening protocol (scenario A, control) and the suggested screening protocol scenarios.

Based on colonoscopy as the second screening stage in all scenarios, seven first screening stage scenarios were developed and used for the analysis:

Scenario A. The primary screening protocol included FITs and the HRFQ (positive standards described in methods). Scenario A has been applied as a national CRC screening protocol in China for years and was used as the control for comparison with other scenarios.
Scenario B. Similar to scenario A but the positive standards for at least two of the following events were removed: a) chronic coprostasis, b) chronic diarrhea, c) phlegmatically bloody feces, d) severely stressful life events among first-degree relatives, e) chronic appendicitis or appendectomy, and/or f) chronic cholecystitis or cholecystectomy in the primary screening.
Scenario C. Similar to scenario A, but the positive standard of a history of colorectal adenoma in the primary screening was removed.
Scenario D. Similar to scenario A, but the positive standard of a history of cancerous events in the primary screening was removed.
Scenario E. Similar to scenario A, but the positive standard of a family history of CRC in first-degree relatives in the primary screening was removed.
Scenario F. Similar to scenario A, but the positive standard of the HRFQ in the primary screening was removed.
Scenario G. Similar to scenario A, but the positive standard of FITs in the primary screening was removed.

Scenarios B, C, D, E, F, and G were used to evaluate the effect of the deleted event in each respective scenario on the screening protocol (i.e., scenarios B, C, D, E, F, and G were also used to evaluate the effects of the reserved subitems on the CRC screening protocol).

Advanced adenoma was defined as an adenoma ≥10 mm or with a history showing either a ≥ 20% villous component or high dysplasia. CRC and advanced adenoma were defined as advanced neoplasm, and CRC, adenoma and polyps were defined as total colorectal neoplasm [14].

Cost estimation: All participants received primary screenings. The HRFQ cost CNY ¥ 5.00 (Renminbi) per case was stratified as follows: ¥ 0.50 for questionnaire printing, ¥ 3.00 for the investigation, ¥ 1.00 for delivery and administration and ¥ 0.50 for participant enrollment. The FIT1 cost CNY ¥ 8.00 (Renminbi) per case was stratified as follows: ¥ 5.00 for the test kits, ¥ 1.50 for sample collection, ¥ 0.50 for the testing fee, and ¥ 1.00 for test organization. The FIT2 cost was ¥ 7.00 per case (the test organization fee was already supported by FIT1). The total cost of the colonoscopy was ¥ 270.00 (42.50 USD) per case. An increase or decrease in one or more questions in the screening protocol, as in scenarios B, C, D, and E, resulted in minimal change compared with scenario A and could thus be ignored. The currency exchange rate between the Renminbi and the US dollar (USD) on August 27, 2012, was ¥ 6.357 for one USD ($1.00).

## Results

A total of 27,076 participants completed the HRFQ, and 24,409 completed at least one FIT. In total, 4075 participants had positive results on the first stage that was eligible for a colonoscopy (defined as the high-risk population and recommended for a colonoscopy). Among them, 3205 participants completed a colonoscopy. The overall compliance rate was 84.7% (27,076/31,963) at the first stage and 78.7% (3205/4075) at the second stage. The compliance rate at the first stage = participants completing either the HRFQ or at least one FIT (*N* = 27,076)/target population (*N* = 31,963). The compliance rate at the second stage = participants completing an endoscopy (*N* = 3205)/high-risk population requiring endoscopy (*N* = 4075).

Scenarios F, G, B and E reduced the high-risk population by 56.8, 35.2, 24.2 and 17.6%, respectively (Table 1). Scenarios C and D reduced the high-risk population by 6.0 and 3.6%, respectively.

**Table 1:** Detection rates in the high-risk population using different screening protocols

	High-risk population/ Reduced population(%)	CRC		Advanced neoplasm		Adenoma		Neoplasm	
		number(%)	OR(95% CI)	number(%)	OR(95% CI)	number(%)	OR(95% CI)	number(%)	OR(95% CI)
Scenario A	4075/0	39(100.0)	1.0	181(100.0)	1.0	359(100.0)	1.0	524(100.0)	1.0
Scenario B	3090/985(24.2)	39(100.0)	1.32(0.85–2.07)	161(89.0)	1.18(0.95–1.47)	308(85.8)	1.15(0.98–1.35)	441(84.2)	1.13(0.98–1.29)
Scenario C	3832/243(6.00)	39(100.0)	1.04(0.66–1.62)	171(94.5)	1.01(0.81–1.24)	340(94.7)	1.01(0.86–1.18)	495(94.5)	1.01(0.88–1.15)
Scenario D	3928/147(3.61)	39(100.0)	1.04(0.66–1.62)	180(99.4)	1.03(0.84–1.28)	358(99.8)	1.04(0.89–1.21)	517(98.7)	1.03(0.90–1.17)
Scenario E	3357/718(17.6)	39(100.0)	1.22(0.78–1.90)	172(95.0)	1.16(0.94–1.44)	327(91.1)	1.12(0.95–1.31)	476(90.1)	1.12(0.98–1.28)
Scenario F	1762/2313(56.8)	39(100.0)	2.34(1.50–3.66)	127(70.2)	1.67(1.32–2.11)	211(58.8)	1.41(1.18–1.69)	296(56.5)	1.37(1.17–1.60)
Scenario G	2642/1433(35.2)	11(28.2)	0.43(0.22–0.85)	78(43.1)	0.65(0.50–0.86)	189(52.3)	0.80(0.66–0.96)	341(65.1)	1.00(0.87–1.16)

OR = (number of cases/high-risk population, scenarios B–G)/(number of cases/high-risk population, scenario A)

Neoplasm including CRC, adenoma and non-adenomatous polyps

The detection rates of CRC, advanced neoplasm, adenoma and colorectal neoplasm in the high-risk population and in the participants that completed the colonoscopy were similar (Tables 1 and 2). The total number of new CRC cases detected by scenarios B–F did not decrease compared with those of scenario A. In scenario F (FITs only without the HRFQ at the first screening stage), the CRC detection rate increased by 1.28–1.34 times that of scenario A. The detection rates of advanced neoplasm, adenoma and neoplasm dramatically improved by 0.63–0.67, 0.37–0.41 and 0.34–0.37 times, respectively, in both the high-risk population and the participants that completed the colonoscopy, although 29.8, 41.2 and 43.5% of advanced neoplasm, adenoma and neoplasm cases were missed. If FITs were removed (scenario G), only 28.2% of CRC cases were detected, and the CRC detection rate decreased significantly (by approximately half) in both the high-risk population (OR = 0.43, 95% confidence interval [CI]: 0.22–0.85) and in participants that completed the colonoscopy (OR = 0.44, 95% CI: 0.22–0.86).

**Table 2:** Detection rates for participants that completed the colonoscopy using different screening protocols

	Colonoscopy participants	CRC		Advanced neoplasm		Adenoma		Neoplasm	
		number (%)	OR (95% CI)	number (%)	OR (95% CI)	number (%)	OR (95% CI)	number (%)	OR (95% CI)
Scenario A	3205	39(100.0)	1.0	181(100.0)	1.0	359(100.0)	1.0	524(100.0)	1.0
Scenario B	2439	39(100.0)	1.32(0.84–2.06)	161(89.0)	1.18(0.95–1.47)	308(85.8)	1.15(0.97–1.35)	441(84.2)	1.13(0.98–1.30)
Scenario C	3002	39(100.0)	1.07(0.68–1.67)	171(94.5)	1.01(0.81–1.25)	340(94.7)	1.01(0.87–1.19)	495(94.5)	1.01(0.88–1.16)
Scenario D	3123	39(100.0)	1.03(0.66–1.61)	180(99.4)	1.02(0.83–1.26)	358(99.8)	1.03(0.88–1.20)	517(98.7)	1.02(0.89–1.16)
Scenario E	2649	39(100.0)	1.21(0.78–1.90)	172(95.0)	1.16(0.94–1.44)	327(91.1)	1.12(0.95–1.31)	476(90.1)	1.12(0.98–1.29)
Scenario F	1430	39(100.0)	2.28(1.45–3.56)	127(70.2)	1.63(1.29–2.06)	211(58.8)	1.37(1.14–1.65)	296(56.5)	1.34(1.14–1.57)
Scenario G	2050	11(28.2)	0.44(0.22–0.86)	78(43.1)	0.66(0.50–0.87)	189(52.3)	0.81(0.67–0.97)	341(65.1)	1.02(0.88–1.19)

OR = (number of cases/participants that completed the colonoscopy, scenarios B-G)/(number of cases/participants that completed the colonoscopy, scenario A)

The ORs of the detection rates of CRC, advanced neoplasm, adenoma, and neoplasm and cost per CRC, advanced neoplasm, adenoma, and neoplasm case in scenarios B–E were near 1.00 (using scenario A as the control) with no significant difference, but the OR and its 95% CI in scenario F were greater than 1.00. However, in scenario G, these values were significantly lower than 1.00 (except for colorectal neoplasm, in which the OR was 1.00 (95% CI: 0.87–1.16) and 1.02 (95% CI: 0.88–1.19) (Tables 1, 2 and 3).

**Table 3:** Costs in both Chinese Renminbi (CNY, ¥) and US dollars ($) using different CRC screening protocols in China, 2007–2009

	CRC			Advanced neoplasm			Adenoma			Neoplasm		
item	Number	Detection cost (¥/$)^a^	cost Ratio*	Number	Detection cost (¥/$)^a^	cost Ratio*	Number	Detection cost (¥/$)^a^	cost Ratio*	Number	Detection cost (¥/$)^a^	cost Ratio*
Scenario A	39	34,409/5413	1.0	181	7414/1166	1.0	359	3738/588	1.0	524	2561/403	1.0
Scenario B	39	29,105/4578	0.846	161	7050/1109	0.951	308	3685/580	0.986	441	2574/405	1.005
Scenario C	39	33,003/5192	0.959	171	7527/1184	1.015	340	3786/596	1.014	495	2600/409	1.015
Scenario D	39	33,841/5323	0.983	180	7332/1153	0.989	358	3687/580	0.986	517	2553/402	0.998
Scenario E	39	30,559/4807	0.888	172	6929/1090	0.935	327	3644/573	0.974	476	2504/394	0.978
Scenario F	39	18,657/2935	0.542	127	5729/901	0.773	211	3448/542	0.922	296	2458/387	0.960
Scenario G	11	62,625/9851	1.820	78	8832/1389	1.191	189	3645/573	0.974	341	2020/318	0.789

^a^The currency exchange rate was 1.000 USD = 6.357 CNY at the time the screening program was performed

Detection cost = cost3/number of cases, cost ratio = detection costs for scenarios B, C, D, E, F and G/detection cost of scenario A

*Cost Ratio= cost of scenario B, or C,or D, or E, or F, or G/ cost of scenario A

**Fig. 1:**
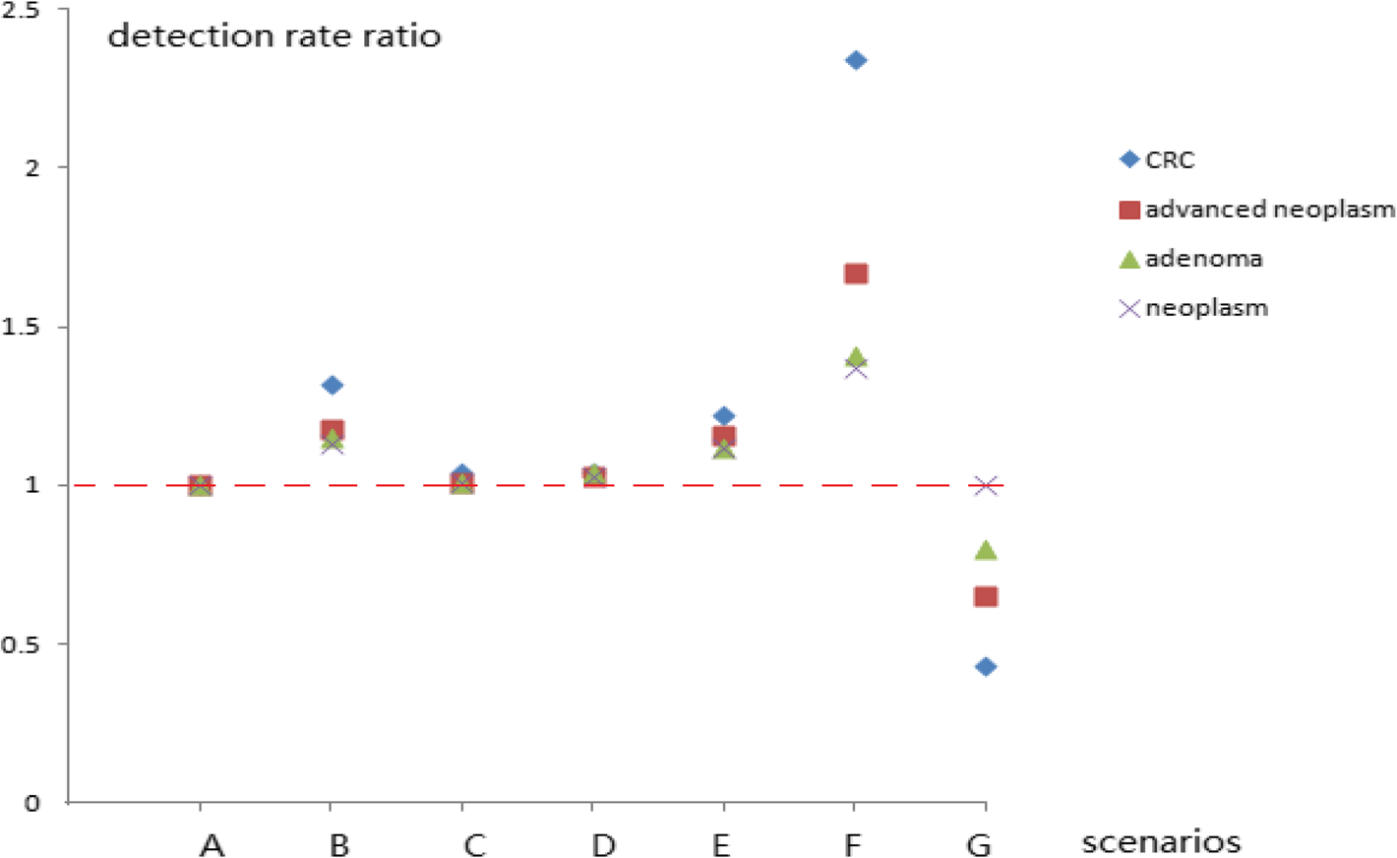
Detection rate ratios of various colorectal neoplasms in the high-risk population using different screening protocols

**Fig. 2:**
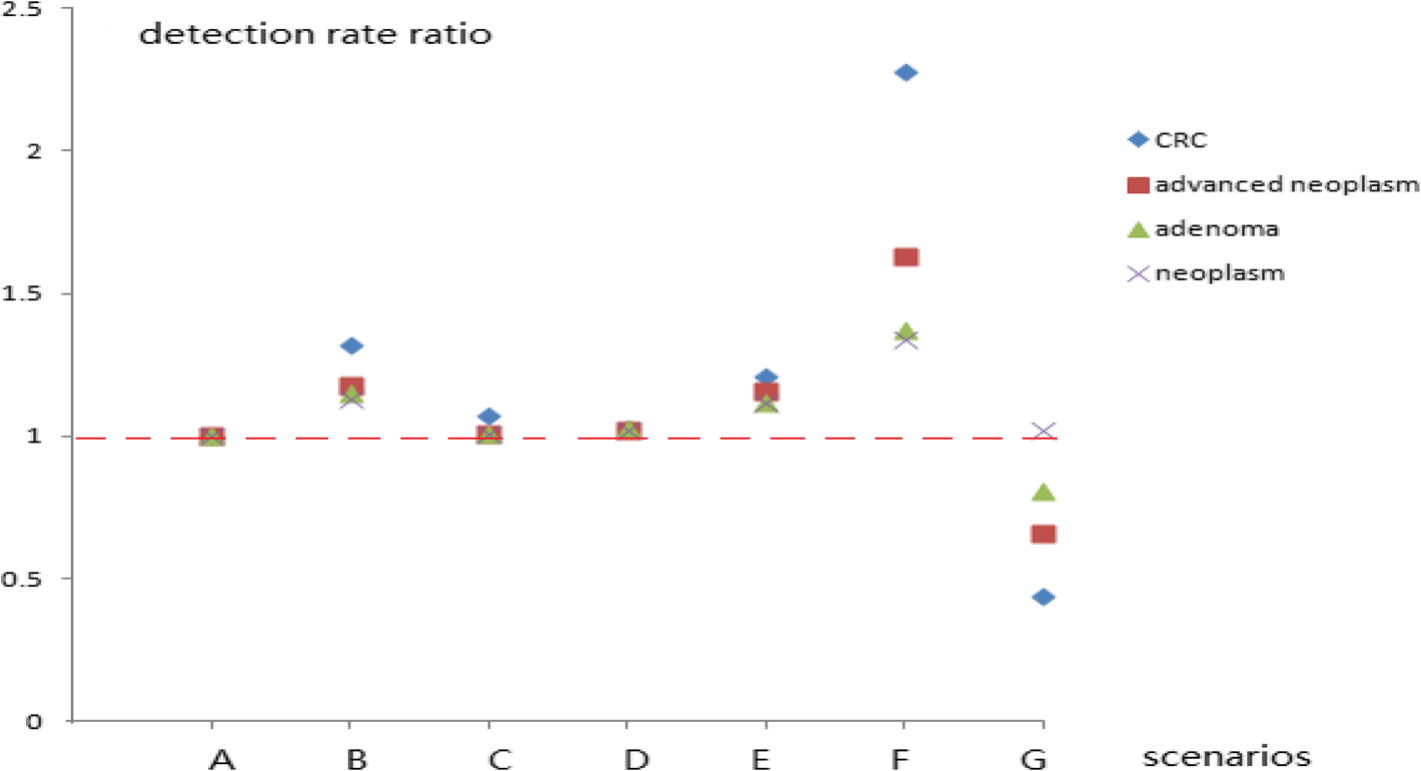
Detection rate ratios of various colorectal neoplasms in participants that completed the colonoscopy using different screening protocols

**Fig. 3:**
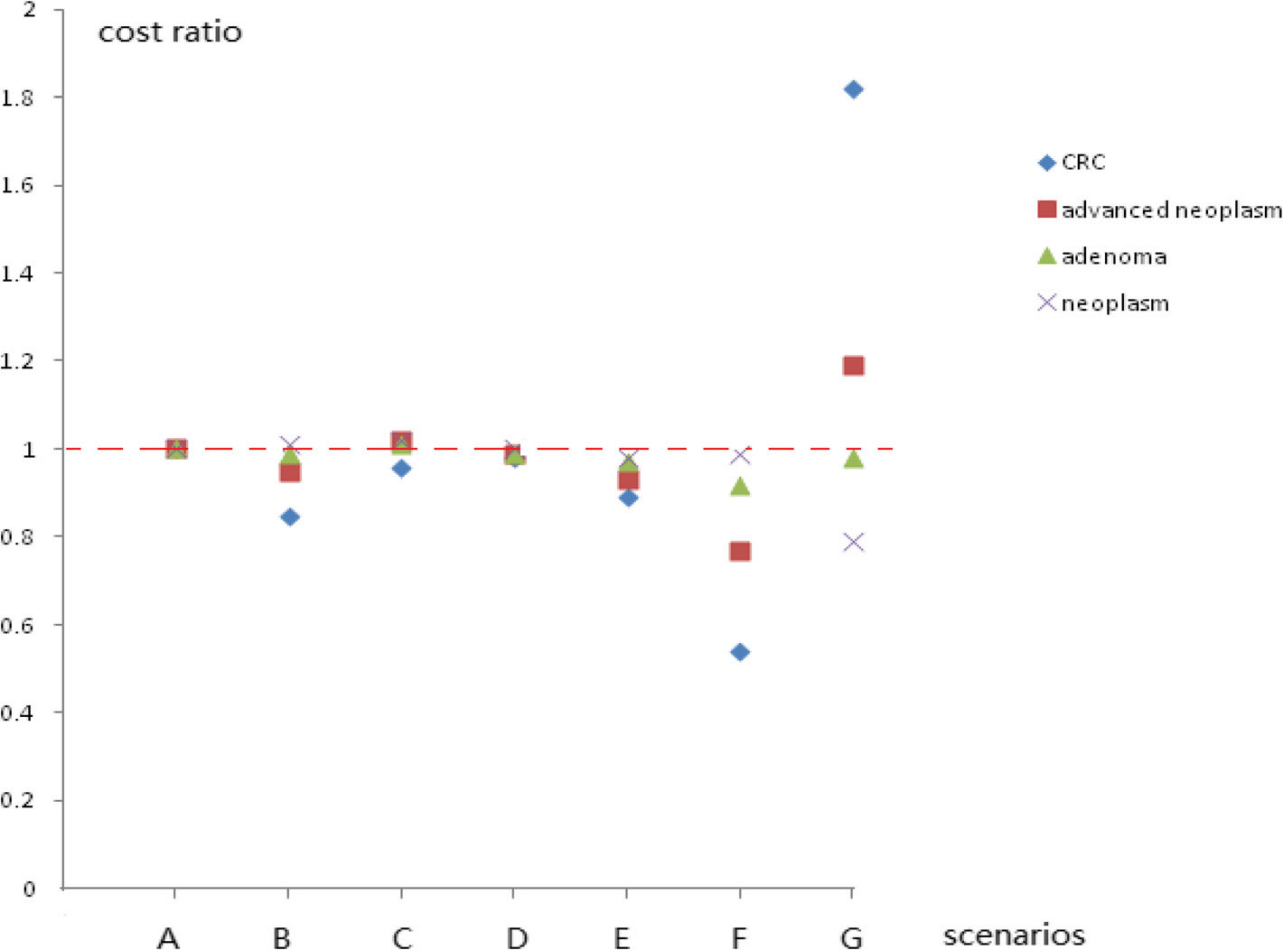
Cost ratios of various colorectal neoplasms using different CRC screening protocols

As shown in Table 4, the cost at the first screening stage and the cost of the colonoscopy at the second screening stage accounted for 35.5% (74,970/211,095 USD) and 64.5% (13,125/211,095 USD), respectively, of the total cost, while the cost of the HRFQ and the FITs accounted for 10.09% (21,296/211,095 USD) and 25.41% (53,673/211,095 USD), respectively, of the total screening cost. The total screening costs of scenarios B–G were reduced by 15.4, 4.1, 1.6, 11.2, 45.8 and 48.7%, respectively, compared with the costs of scenario A. As shown in Table 3, the detection cost ratios of scenarios B–G were used to evaluate the cost-effectiveness using scenario A as the control. The detection costs for CRC, advanced neoplasm, adenoma and all colorectal neoplasms in scenarios B–E were similar to that of scenario A (the OR was near 1.0). Scenario F significantly reduced the detection costs of CRC and advanced neoplasm by 45.8 and 22.7%, respectively, while scenario G increased the detection costs by 82.0 and 19%, respectively. However, the detection costs of adenoma and all colorectal neoplasms (scenarios F and G, respectively) were near those of scenario A (OR ≥ 0.92), except that the OR in scenario G for colorectal neoplasm was 0.79 compared with that of scenario A (Table 3, Figs. 1, 2 and 3).

**Table 4:** Numbers of participants (including FIT-positive and those that completed the colonoscopy) and relevant costs in the Jian-Shan mass CRC screening program in China, 2007–2009

Item	Participants	Cost1 (¥/ $)^a^	Positive participants	Participants completing colonoscopy	Cost2 (¥/ $)^a^	Cost3 (¥/ $)^a^	% (of scenario A)
FIT1	24,375	195,000/30,675	1148	926	250,020/39,330	445,020/70,005	
FIT2	20,886	146,202/22,999	915	754	203,580/32,025	349,782/55,023	
FIT Either	24,419	341,202/53,673	1762	1430	386,100/60,736	727,610/114,458	
HRFQ	27,076	135,380/21,296	2642	2050	553,500/87,069	688,880/108,366	
Total	27,076	476,582/74,970	4075	3205	865.350/136,125	1,341,932/211,095	
Scenario A	27,076	476,582/74,970	4075	3205	865.350/136,125	1,341,932/211,095	100
Scenario B	27,076	476,582/74,970	3090	2439	658,530/103,591	1,135,112/178,561	84.6
Scenario C	27,076	476,582/74,970	3832	3002	810,540/127,504	1,287,122/202,473	95.9
Scenario D	27,076	476,582/74,970	3928	3123	843,210/132,643	1,319,792/207,612	98.4
Scenario E	27,076	476,582/74,970	3357	2649	715,230/112,511	1,191,812/187,480	88.8
Scenario F	24,419	341,202/53,673	1762	1430	386,100/60,736	727,610/114,458	54.2
Scenario G	27,076	135,380/21,296	2642	2050	553,500/87,069	688,880/108,366	51.3

Abbreviations: FIT1, the first FIT; FIT2, the second FIT; FIT2 refers only to those who completed FIT2 without completing FIT1

^a^ The currency exchange rate was 1.000 USD = 6.357 CNY at the time the screening program was performed

Cost1 refers to the cost for completing the FITs and/or the HRFQ. Cost2 = colonoscopy participants*270 yuan (RMB), the cost of completing the colonoscopy

Cost3 = cost1 + cost2, the total screening cost covering the first and second stages

Some advanced adenoma appears at early age such as 40–49 years old. There were 12.15%(22/181,including 2 CRC cases) advanced neoplasm cases aged 40–49 detected in this screening program.

## Discussion

In China in 2015, approximately 3.736 million new CRC cases apeared, and 1.91 million patients with CRC died [2]. CRC incidence and mortality is expected to continue to increase for a long time [2, 15]. Finding an easy method for decreasing CRC incidence and mortality is difficult. However, several randomized controlled trials in China and Western countries have shown that CRC screening can effectively reduce CRC morbidity and mortality [3–6]. Because patients with advanced colorectal adenoma are at a high risk of developing CRC, the CRC screening protocol should be targeted at both CRC and advanced adenoma (having both CRC and advanced adenoma indicates advanced colorectal neoplasm). Thus, the endpoint of our screening was not only CRC but also advanced colorectal adenoma, and the colorectal tumor prevention strategies should move from cancer to precancerous lesions, and advanced colorectal neoplasms will play increasingly important roles in future CRC screening protocols [16, 17].

Colonoscopies are a good CRC screening method in some countries; however, they are expensive and impractical in China because they require experienced doctors in urban areas. No perfect protocol exists for CRC screening that can be applied globally [10, 11, 18]. The protocol that combines the HRFQ and FIT has been a practical and cheap CRC screening protocol in China for years. However, this protocol has some problems and causes much of the high-risk population to be screened out in the first screening stage, and participants in the second stage must undergo colonoscopies, thus resulting in high costs [12]. Therefore, this protocol should be evaluated in detail by determining the detection rates of CRC and advanced colorectal neoplasm as well as the cost-effectiveness of the protocol [19]. Based on this screening protocol, this study developed seven screening scenarios, which were used to determine the most cost-effective and practical CRC screening methods.

Accuratly evaluating CRC screening protocol require a high screening compliance [19]. Fortunately, the CRC screening compliance rate was satisfied in the analyzed communities (84.7% at the first stage and 78.7% colonoscopy at the second stage), which enables obtaining more reliable results in the mass CRC screening program.

Age and sex are very important in the CRC screening, and we had enrolled both males and females and the high-risk age population of 40- to 74-year-old residents to be the target populations. In this study, we focus mainly on the subitems in the colorectal cancer screening protocol on the Chinese colorectal cancer screening program, the effects of the total Chinese colorectal cancer screening program had been discussed in detail in the previous article [12].

In this study, the high-risk population was reduced by more than 17.6% in scenarios B, E, G, and F and by less than 6.0% in scenarios C and D. Removing FITs (scenario G) reduced the numbers of detected cases of CRC, advanced neoplasm and adenoma to 28.2, 43.1 and 52.3% in the high-risk population, respectively, compared with scenario A, suggesting that FITs is important in the screening protocol and cannot be ignored or removed. This result was verified by other reports including RCT results [20–22]. Because the quantitative FIT test is expensive, it is not practical for CRC screenings in China. The qualitative FIT was used for its availability and cheaper cost; it is also more easily applied in the countryside in China as a developing country. In our opinion, based on the results, if the cost of double FITs is acceptable, and the compliance of the second FIT is not low, double FITs are preferred because they can detect more advanced colorectal neoplasms [23].

In this study, removing the whole HRFQ from the screening protocol (scenario F), reduced the detected cases of advanced neoplasm and adenomas by 29.8 and 41.2%, respectively, compared with scenario A, whereas the whole HRFQ accounted for only 10.09% (135,380/1,341,932) of the total CRC screening cost (Table 4). This result indicates that the HRFQ may be more cost-effective in the CRC screening protocol and cannot be removed entirely [12, 24, 25]. We needed to determine which question(s) on the HRFQ were more important and more cost-effective in the mass CRC screening program; therefore, scenarios B–E were developed to analyze the effectiveness of the questions.

In the current study, if any one high-risk factor item on the HRFQ was removed, such as in scenarios B–E, the CRC, advanced neoplasm, adenoma, and neoplasm detection rates did not differ significantly from those of scenario A (Tables 1 and 2). Tables 1, 2, 3 and 4 and Figs. 1, 2 and 3 show that the detection rates for CRC and advanced neoplasm, as well as the screening cost per CRC and advanced neoplasm case (scenarios B–E) did not vary significantly from those of scenario A. From a health economist’s viewpoint, a CRC screening protocol should reduce the screening costs by decreasing the number of participants requiring colonoscopies if the number of detected advanced colorectal neoplasms is small and if it is cost-effective in screening for CRC or advanced colorectal neoplasm. Therefore, scenarios E and B are preferred because they reduced the numbers of participants requiring colonoscopies by 17.6% (718) and 24.2% (985), respectively, and screening costs by 14.5% (reducing the number of patients requiring a colonoscopy*colonoscopy examination fee/total screening cost, 718*42.5/211,095 USD) and 19.8% (985*42.5/211,095 USD), respectively. Conversely, the numbers of detected advanced colorectal neoplasms decreased by only 5.0 and 11.0%, respectively (Table 1). This result indicates that personal histories of cancer or colorectal adenoma (which were included in scenarios B and E) should be prioritized in the HRFQ in the screening protocol. According to the American National Comprehensive Cancer Network (NCCN) guidelines for detecting, preventing, and risk reduction of CRC, patients with personal histories of colorectal adenoma are considered at an increased risk and are recommended for a colonoscopies every 3–10 years (https://www.nccn.org/professionals/physician_gls/default.aspx#colorectal_screening) based on the number of adenomas and the pathological classification [17, 18, 20].

Scenario E removed the positive for family history of CRC question and showed good cost-effectiveness. In China, the CRC incidence is low in the natural population (20–60/100,000), and less than 15% of CRC patients have family histories of CRC, indicating that using a family history of CRC to screen out CRC and advanced colorectal neoplasm may be less cost-effective in the natural community.

In a previously published article [12], we analyzed scenarios A, F, and G by sex and age. Few CRC cases were detected (single digit cases for any removed question, total less than 28.2%, 11/39) by reducing the number of questions suggested in scenarios B–E. Exploring scenarios B–E by sex and age would require a larger sample size, and we plan to do this in future studies.

Based on the detection rates of CRC and advanced neoplasm and the cost-effectiveness analysis, FITs and a personal history of colorectal adenoma are the most effective items in the CRC screening protocol, and the HRFQ has some cost-effectiveness in the CRC screening protocol and thus cannot be removed or ignored entirely. Other items on the HRFQ adopted in the CRC screening protocol, except personal history of cancer and colorectal adenoma, require additional evidence to support their necessity and effectiveness.

## Abbreviations

CRC: Colorectal cancer
FITs: Fecal immunochemical tests
FOBT: Fecal occult blood test
HRFQ: High-risk factor questionnaire
NCCN: National Comprehensive Cancer Network.
ORs: Odds ratios
RCTs: Randomized control trials
